# Editorial: Women in psychiatry 2021: Forensic psychiatry

**DOI:** 10.3389/fpsyt.2022.1051071

**Published:** 2022-10-11

**Authors:** Hedvig Krona, Birgit Völlm, Katarina Howner

**Affiliations:** ^1^Department of Psychiatry and Neurochemistry, Centre for Ethics, Law and Mental Health, Institute of Neuroscience and Physiology, The Sahlgrenska Academy at University of Gothenburg, Gothenburg, Sweden; ^2^The Regional Forensic Psychiatric Clinic, Växjö, Sweden; ^3^Department of Clinical Sciences Lund, Child and Adolescent Psychiatry, Faculty of Medicine, Lund University, Lund, Sweden; ^4^Clinic and Outpatient Department of Forensic Psychiatry, University Medicine Rostock, Rostock, Germany; ^5^Department of Clinical Neuroscience, Centre of Psychiatry Research, Karolinska Institutet, Solna, Sweden; ^6^Division for Forensic Psychiatry in Stockholm, Department for Forensic Psychiatry, National Board of Forensic Medicine, Stockholm, Sweden

**Keywords:** women in forensic psychiatry, female offenders, gender, women in science, female scientists, forensic psychiatry

Throughout modern times there has been a significant gender gap at all levels of science, technology, engineering and mathematic (STEM) disciplines, and only 33% of all researchers around the world are women according to the UNESCO Science report (1). In the 2030 Agenda for Sustainable Development (2) the United Nations identified that science and gender equality are vital for the achievement of the internationally agreed development goals. Furthermore, diversity and gender equality are important aspects to enhance in the continuous work in improving excellence and quality in science. By encouraging more female scientists to pursue research careers, particularly in STEM, the possibility to defeat stereotypic views of both scientists and research subjects emerges. This is particularly important vis-à-vis the fields of psychiatry and forensic psychiatry, as there is an added stigma connected to tenacious prejudices about sufferers of mental health disorders. This collection of articles by female main authors covers a range of topics related to the subject of women in forensic psychiatry.

One group of articles addresses issues of gender directly. Ali and Adshead provide an overview of gender as a social construct and explore how gender role stereotypes impact upon how psychological distress is communicated by men and women, and the relationship between violence, gender and mental health. In their article, women as violent offenders, as patients in secure psychiatric facilities but also as clinicians in forensic settings are examined. The authors focus on whether patriarchal influences and gender role stereotypes may have had an impact on the development of women's forensic mental health services. They caution us to be aware of gender as a social construct in forensic services in order to not cause harm to our patients. Neatly related to this topic, Joyes and Jordan analyse ethnographic data from a forensic mental health hospital in the UK. The over 300 h of fieldwork provide a unique insight into the communication between staff and patients, demonstrating the presence of misogynistic everyday talk between staff. The authors argue that such attitudes are part of a continuum including a permissive mindset towards gender-based violence, and that they are particularly problematic in the context of patients sentenced for similar offences.

A number of papers address characteristics of women as offenders and victims.

Hodgins reviews descriptive studies of females sentenced to forensic psychiatric treatment and shows that most female aggressive and antisocial behaviour does not lead to criminal prosecution. Furthermore, the article highlights known facts about the two most common mental disorders in female forensic patients, namely schizophrenia and borderline personality disorder. Finally, Hodgins provides recommendations for earlier identification of women presenting with both mental disorders and aggressive and antisocial behaviour in psychiatric services.

Streb et al. present a study of male and female forensic psychiatric patients in the German forensic psychiatric system, examining the presence of substance use disorders and comparing socio-demographic, legal and clinical characteristics between the sexes. Differences were found in all these domains. The authors identify sex-specific characteristics, in particular past trauma, that should be considered in forensic psychiatric therapy. In Sweden, Caman et al. study clinical characteristics of perpetrators of intimate partner femicide (IPF) in comparison to male to male homicide (MMH) perpetrators, and find a higher proportion of individuals with substance use disorders in the MMH group compared to the IPF group. The proportion of homicide-suicide was relatively common in the IPF group (20%), suggesting that previous suicide attempts and suicide ideation might be important indicators for predicting and possibly preventing IPF.

Our collection also includes papers which do not address gender issues directly but demonstrate significant scientific contributions by female authors.

Markham offers a theoretical piece exploring the totalising and risk aversive nature of secure forensic mental health services. She looks in particular at restrictive practices and practitioner attitudes, and how they can cause iatrogenic harm and thereby hindering healing and recovery.

In their perspective article, Lennox et al. reflect on how randomized controlled trials (RCT), known as the gold standard for measuring the effectiveness of an intervention, have limitations particularly in prison settings. The authors share their experiences by describing two of their RCTs and through that propose that this particular research design may limit the understanding and ability to test complex interventions in prison settings. In lieu of RCTs, the authors call for more flexible and adaptive study designs. In another perspective article by Kip and Bouman the authors discuss how eHealth interventions could improve forensic psychiatric care, but that the uptake in practice is low. They explore how possibilities for eHealth could be connected to the risk-need-responsivity (RNR) model, where stand-alone eHealth interventions might be used to offer more intensive treatment to high-risk offenders. Novel experience-based interventions such as virtual reality (VR) and apps could also be made an integral part of forensic-psychiatric treatment. Furthermore, Göranson et al. report on a rarely studied subject. By using case vignettes presented to three professional groups, the authors investigate which types of information experts use to reach conclusions on legal insanity. Understanding the process is important in order to counteract potential bias which may include gender bias, though this is not the topic of the authors' contribution.

A group of papers include research on forensic psychiatric patients, investigating life time criminality, treatment process and the occurrence of self-harm during in-patient treatment. In their research article utilising a total cohort of forensic psychiatric patients in a Swedish setting, Krona et al. explore the possibility of a sub-group of particularly criminality prone individuals. Through statistical analysis, a small group defined by childhood adversities, neurodevelopmental disorders and later substance use emerged. The study replicates findings from prison populations, showing that there is a sub-group of individuals sharing early-onset disorders, childhood adversities and substance use disorders, who are more criminally persistent. Jankovic et al. present in their study of a nationwide sample of Dutch forensic psychiatric patients various long-term changes in dynamic and protective factors. The authors investigate trajectories of risk and protective factors over time in all 722 male forensic psychiatric patients who were unconditionally released between 2004 and 2014. Findings indicate that all changes in dynamic risk and protective factors could be depicted in two phases of the patients stay: the beginning of the stay and at the transition to unsupervised leave, which could be considered a turning point in the treatment. Jeandarme et al. analyse characteristics of discharged and not discharged (long-term) forensic patients in two newly implemented forensic high security settings in Flanders by studying files of an admission cohort of 654 patients. Their conclusion is that the Flemish forensic patients are characterized by a high proportion of sex offenders and personality disorders. The group of patients with personality disorders, especially those with elevated psychopathic traits, remain longer than expected and are more difficult to re-socialize. Laporte et al. investigate the important topic of self-harm, which has a much higher prevalence rate in forensic mental health settings compared to the general population and is one of the leading causes of death in these settings. The authors look at the clinical needs of individuals who self-harmed in forensic mental health settings in Sweden over a five-year period. Two thirds of their sample had self-harmed at some point with the most common method being head banging, banging one's fist against a solid surface and cutting. Self-harm was often associated with self-punishment and difficulties regulating affect.

Finally, Lutz et al. evaluate a specialized ward for language acquisition in a German forensic-psychiatric hospital, and found that patients on this ward achieve significantly better German language skills compared to regular wards, with literacy being an important predictor. The authors argue that more effort needs to be made to support language acquisition in order to enable patients to participate in treatment more effectively.

## Author contributions

HK, BV, and KH wrote the manuscript and contributed to the final version. All authors read and approved the submitted version.

## Conflict of interest

The authors declare that the research was conducted in the absence of any commercial or financial relationships that could be construed as a potential conflict of interest.

## Publisher's note

All claims expressed in this article are solely those of the authors and do not necessarily represent those of their affiliated organizations, or those of the publisher, the editors and the reviewers. Any product that may be evaluated in this article, or claim that may be made by its manufacturer, is not guaranteed or endorsed by the publisher.
